# Cigarette smoke induces epithelial to mesenchymal transition and increases the metastatic ability of breast cancer cells

**DOI:** 10.1186/1476-4598-12-90

**Published:** 2013-08-06

**Authors:** Francescopaolo Di Cello, V Lynn Flowers, Huili Li, Briana Vecchio-Pagán, Brent Gordon, Kirsten Harbom, James Shin, Robert Beaty, Wei Wang, Cory Brayton, Stephen B Baylin, Cynthia A Zahnow

**Affiliations:** 1 Department of Oncology, The Sidney Kimmel Comprehensive Cancer Center at Johns Hopkins, Baltimore, MD 21287, USA; 2 Department of Molecular and Comparative Pathobiology, The Johns Hopkins University School of Medicine, Baltimore, MD 21205, USA

**Keywords:** Tobacco, Breast cancer, Cell motility and invasion, Epithelial to mesenchymal transition, Metastasis, Intraductal injection

## Abstract

**Background:**

Recent epidemiological studies demonstrate that both active and involuntary exposure to tobacco smoke increase the risk of breast cancer. Little is known, however, about the molecular mechanisms by which continuous, long term exposure to tobacco smoke contributes to breast carcinogenesis because most previous studies have focused on short term treatment models. In this work we have set out to investigate the progressive transforming effects of tobacco smoke on non-tumorigenic mammary epithelial cells and breast cancer cells using *in vitro* and *in vivo* models of chronic cigarette smoke exposure.

**Results:**

We show that both non-tumorigenic (MCF 10A, MCF-12A) and tumorigenic (MCF7) breast epithelial cells exposed to cigarette smoke acquire mesenchymal properties such as fibroblastoid morphology, increased anchorage-independent growth, and increased motility and invasiveness. Moreover, transplantation experiments in mice demonstrate that treatment with cigarette smoke extract renders MCF 10A cells more capable to survive and colonize the mammary ducts and MCF7 cells more prone to metastasize from a subcutaneous injection site, independent of cigarette smoke effects on the host and stromal environment. The extent of transformation and the resulting phenotype thus appear to be associated with the differentiation state of the cells at the time of exposure. Analysis by flow cytometry showed that treatment with CSE leads to the emergence of a CD44^hi^/CD24^low^ population in MCF 10A cells and of CD44^+^ and CD49f ^+^ MCF7 cells, indicating that cigarette smoke causes the emergence of cell populations bearing markers of self-renewing stem-like cells. The phenotypical alterations induced by cigarette smoke are accompanied by numerous changes in gene expression that are associated with epithelial to mesenchymal transition and tumorigenesis.

**Conclusions:**

Our results indicate that exposure to cigarette smoke leads to a more aggressive and transformed phenotype in human mammary epithelial cells and that the differentiation state of the cell at the time of exposure may be an important determinant in the phenotype of the final transformed state.

## Background

Multiple epidemiological studies have established the association between active and involuntary exposure to tobacco smoke and increased risk of breast cancer. The link, which has been a controversial topic for many years, was initially demonstrated in younger, primarily premenopausal women [1,2], and subsequently in postmenopausal women [2-4]. The epidemiological evidence is backed up by several studies showing that tobacco carcinogens are present and active in the breast tissue of smokers [1,5-7]. Except for the documented formation of mutagenic DNA adducts [6,8], it is unclear how these compounds affect cell behavior in the breast contributing to cancer development, progression, and metastasis. Emerging evidence suggests that cigarette smoke condensate (CSC), or aqueous cigarette smoke extract (CSE) can induce changes in morphology and gene expression indicative of epithelial to mesenchymal transition (EMT) in immortalized human bronchial epithelial cells [9] and in lung carcinoma cells [9,10]. This implies the acquisition of mesenchymal properties, including traits that are associated with malignancy such as increased motility and invasiveness [11]. Although these studies provide some mechanistic data on tobacco smoke tumorigenesis in lung, data for breast cancer are limited. In this work we have set out to investigate the progressive transforming effects of tobacco smoke on non-tumorigenic mammary epithelial cells and breast cancer cells using *in vitro* and *in vivo* models. Our results indicate that exposure to cigarette smoke leads to a more aggressive and transformed phenotype in human mammary epithelial cells, and that the differentiation state of the cell at the time of exposure may be an important determinant in the phenotype of the final transformed state.

## Results

### Cigarette smoke induces anchorage-independent cell growth, migration, invasion and morphological changes in mammary epithelial cells and breast cancer cells

**Figure 1:**
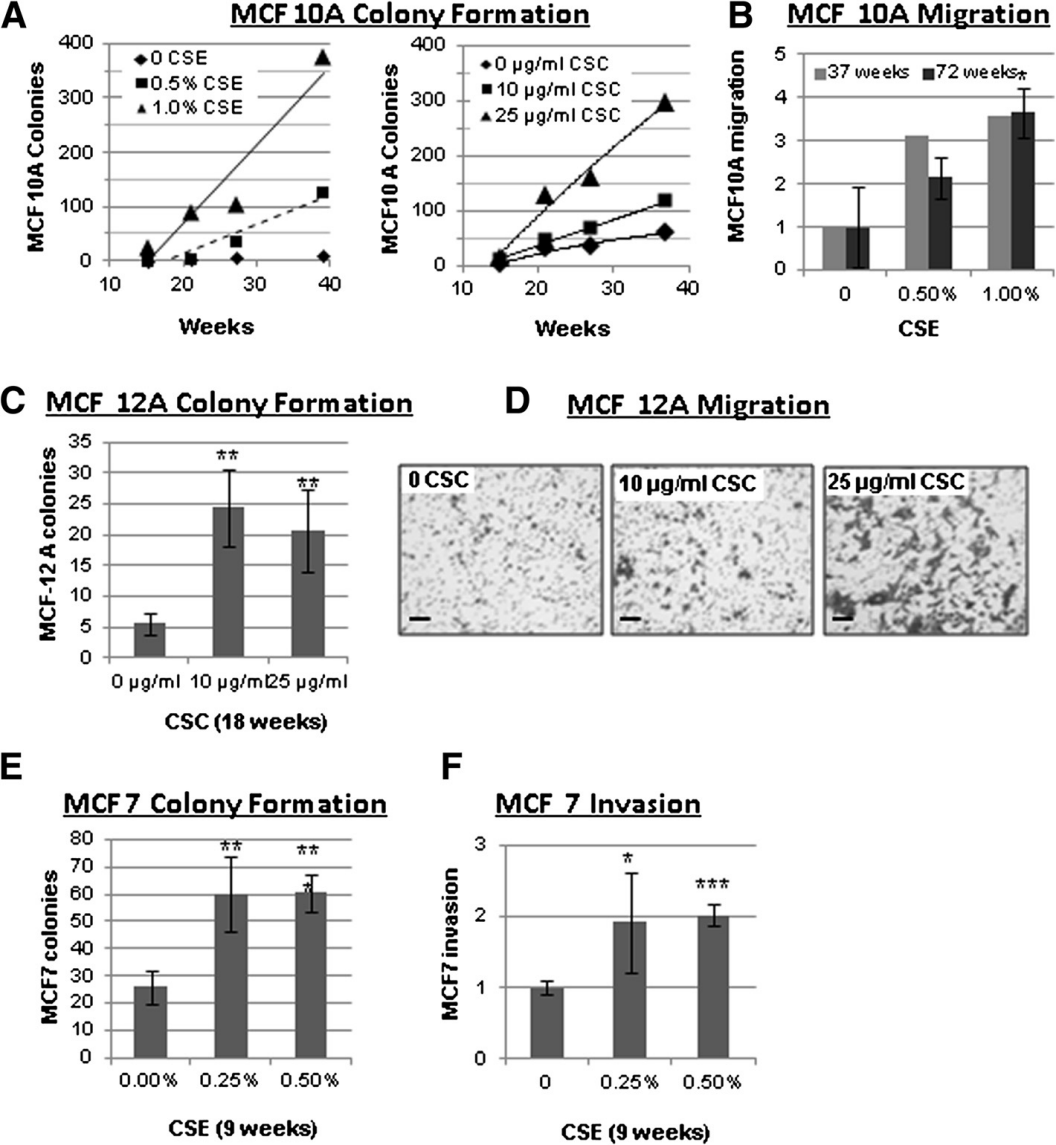
**Cigarette smoke induces anchorage-independent cell growth, migration, and invasion in mammary epithelial cell lines. (A)** Treatment of MCF10A cells with CSE or CSC leads to soft agar colony formation that increases, as compared to mock, with increased weeks of exposure. **(B)** Treatment of MCF10A cells with CSE for 37 and 72 weeks leads to increased migration through uncoated transwell inserts as compared to mock treated cells. **(E)** Treatment of MCF 7 cells with CSE for 9 weeks leads to soft agar colony formation as compared to mock. **(F)** Treatment of MCF 7 cells with CSE for 9 weeks leads to increased invasion through matrigel-coated transwell inserts as compared to mock. **(C)** Treatment of MCF12A cells with CSC for 18 weeks leads to increased colony formation as compared to mock. **(D)** Treatment of MCF12 A cells with CSC leads to increased migration through uncoated transwell inserts as compared to DMSO control The increase in MCF-12A migration (right) was not quantified numerically because the cells became partially confluent after migration and could not be accurately counted. The dark patches are cells stained with crystal violet that have migrated though the filter pores (light grey dots); the size bar represents 100 μm. Data in bar graphs are mean ± standard deviation of 3–5 replicates; *P<0.05, **P<0.01, ***P<0.001 by Student’s *t*-test.

**Figure 2:**
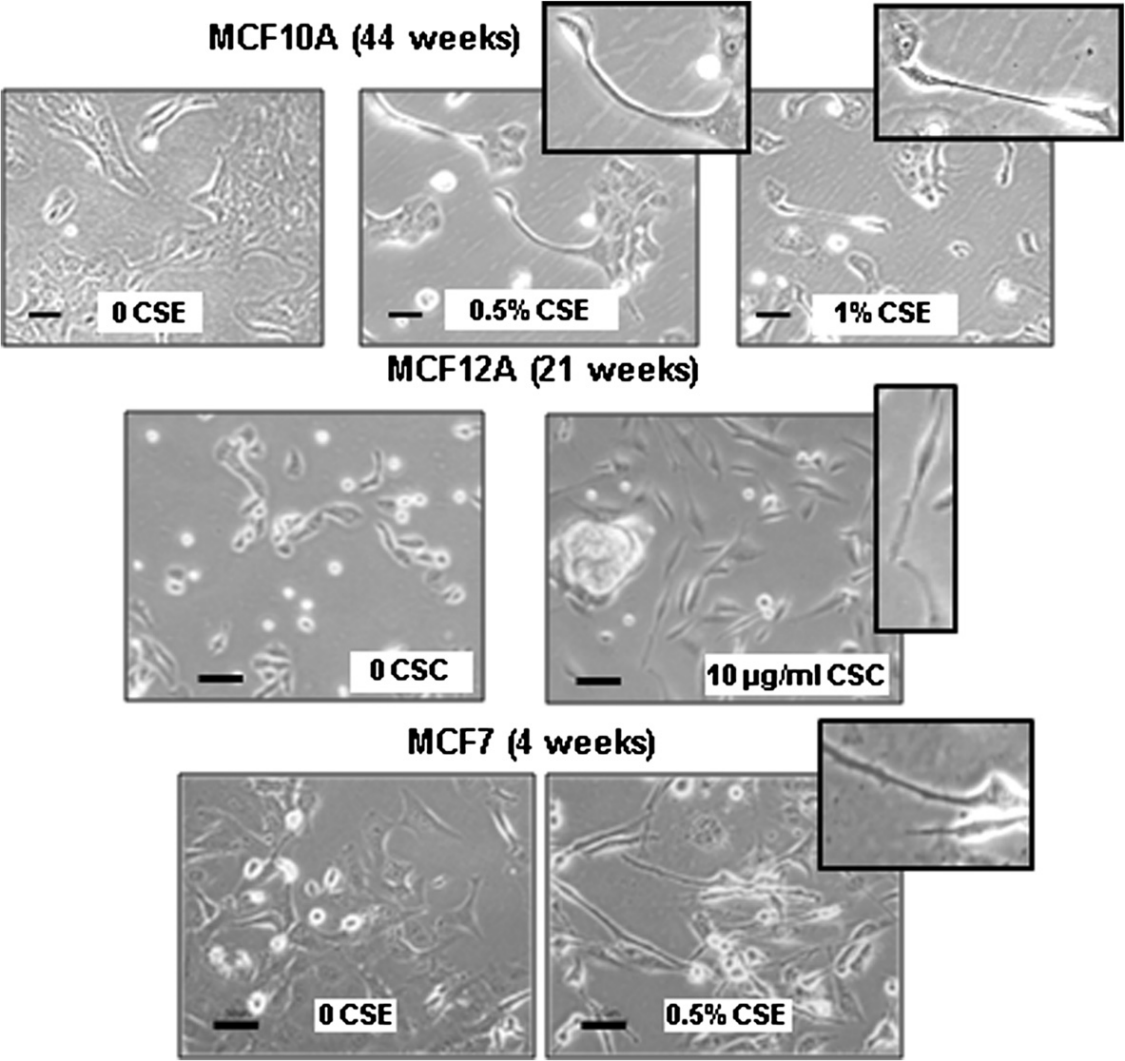
**Cigarette smoke induces morphological alterations in mammary epithelial cell lines.** Photomicrographs showing morphological alterations of MCF 10A cells exposed to CSE for 44 weeks, MCF-12A cells exposed to CSC for 21 weeks, and MCF7 cells exposed to CSE for 4 weeks. Inserts highlight the spindle shape of cells treated with CSE or CSC. The size bar represents 50 μm.

It has been shown that the risk of developing cancer increases with the number of years a person has smoked or been exposed to second hand smoke [12,13]. For this reason we developed a model to study the progressive, chronic effects of cigarette smoke exposure. Cells were continuously cultured for 72 weeks with an aqueous cigarette smoke extract (CSE) from main stream smoke prepared in our laboratory (0.25%, 0.5% or 1% CSE) or for approximately 40 weeks with cigarette smoke condensate (CSC) a commercial product based on condensate from second-hand-like smoke (10 μg/ml or 25 μg/ml CSC). A concentration of 0.5% CSE, or 25 μg/ml CSC in the media corresponds to approximately 0.001 cigarettes/ml, which is an amount comparable to, or lower than those used in other studies [9,10,14-16]. The corresponding amount of nicotine in the media (1.3±0.1 μg/ml) approximates the upper limit of the concentrations of cotinine found in the plasma or breast milk of smokers, which has been reported as high as 300–800 ng/ml and 200–500 ng/ml, respectively [17]. Non-tumorigenic MCF 10A cells cultured with either CSE or CSC were transferred to soft agar to assess anchorage-independent growth after 15, 21, 27 and 39 or 37 weeks of treatment. Both CSE and CSC caused a significant increase in colony formation in soft agar (up to 42 fold; Figure 1A) which is a feature typical of cancer cells. Linear regression analysis indicated that the effect was both dose and time dependent as the number of colonies increased in parallel with the duration of treatment (r^2^>0.9; *P*<0.05 by F-test), and the concentration of CSE or CSC (*P*<0.01 by F-test). The vehicle control for CSC, which contains DMSO, led to the development of slightly more colonies than the saline control for CSE. Treatment with CSE also increased the migratory ability in MCF10As. After treatment with 0.5% or 1% CSE for 37 weeks MCF 10A cells showed a 3.1 and 3.6 fold increase in migration, respectively (Figure 1B). The experiment was replicated after 72 weeks of treatment with similar results, suggesting that the initial increase in motility is maintained during prolonged exposure to CSE (Figure 1B). To test whether colony formation and migration were unique to MCF10As or would also occur in other breast cell lines we treated non-tumorigenic, MCF-12A cells with CSC and the breast cancer cell line MCF7 with CSE. For MCF12A, we observed a 4 to 5-fold increase in colony formation after 18 weeks of treatment (Figure 1C, *P*<0.01 by Student’s *t*-test) and a significant increase in migration when exposed to CSC for 18 weeks (Figure 1D). MCF7 cells are capable of forming colonies without CSE treatment; however, a significant increase in colony formation was observed after only nine weeks of treatment with 0.5% CSE (Figure 1E). Moreover MCF7 cells, which are more motile then MCF 10A and -12A, and have the ability to migrate through matrigel coated filters, showed a marked increase of their invasive capability when exposed to 0.25% or 0.5% CSE for 9 weeks (Figure 1F). All cell lines tested altered their morphology when exposed to CSE or CSC. Untreated MCF 10A and MCF-12A cells normally display a typical cobblestone epithelial morphology in culture. Treatment with CSE or CSC caused them to adopt a more spindle shape and fibroblast-like morphology (shown clearly in the inserts, upper and middle panels, Figure 2), which is consistent with the increased motility that we observed in the migration assays shown in Figure 1. Similarly, MCF7 cells also became more elongated and spindle-shaped after exposure to cigarette smoke (Figure 2, bottom panel). The observed changes in morphology and motility are consistent with phenotypical changes associated with EMT, and suggest that chronic exposure to cigarette smoke may cause breast epithelial cells to acquire mesenchymal properties, which would render them more motile [11]. For cells that are already tumorigenic, such as MCF7, our observations suggest that the phenotype has become more invasive. Similar results were observed using either CSE or CSC, indicating that both mainstream smoke and second hand cigarette smoke contain compounds that can significantly alter the phenotype of these diverse cell lines.

### CSE confers the ability to colonize mammary ducts and metastasize to mammary epithelial cells and breast cancer cells, respectively

**Figure 3:**
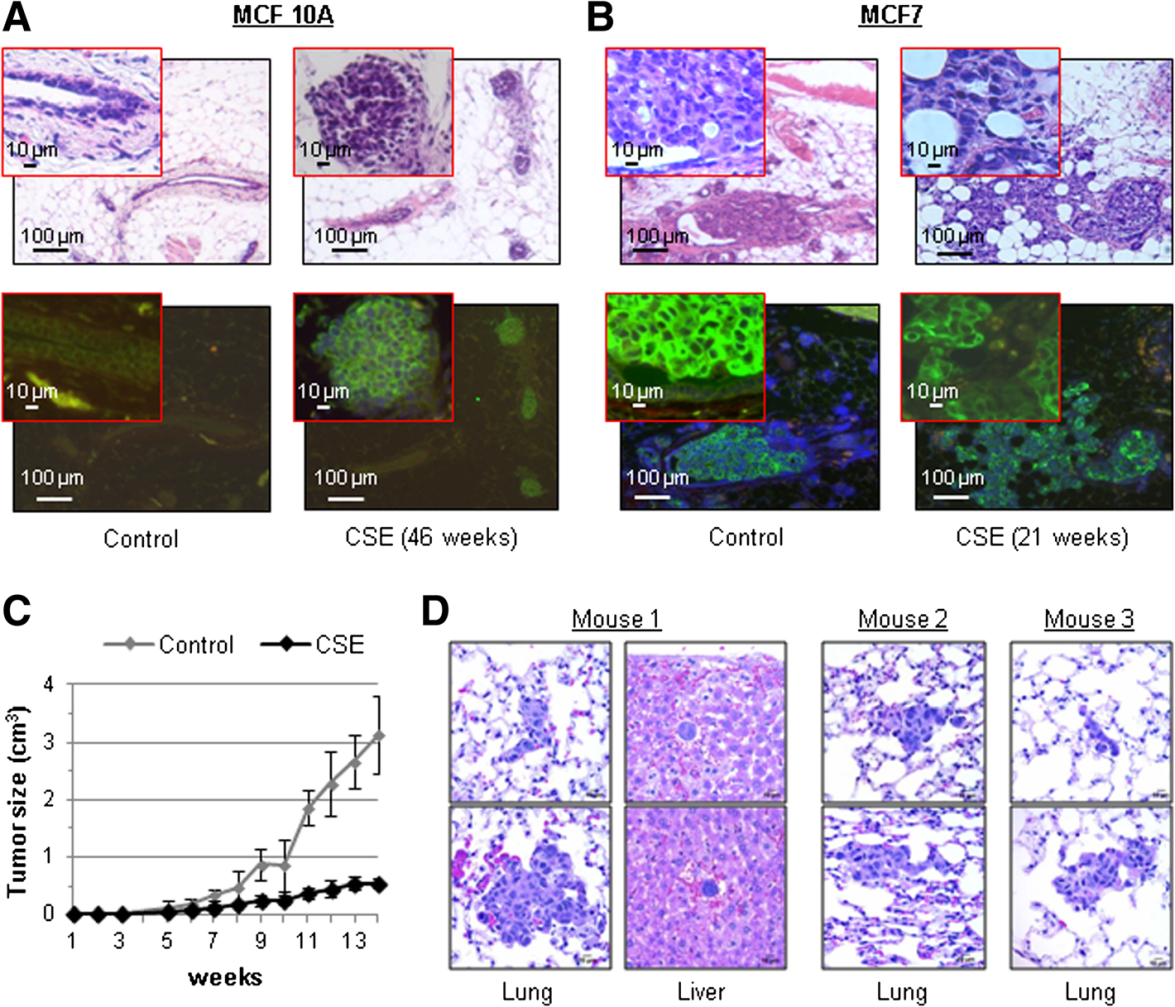
**Cigarette smoke facilitates intraductal colonization of MCF 10A cells and distant metastasis of MCF7 cells. (A-B)** H&E and FITC/DAPI staining of mammary gland sections from NSG immunodeficient mice injected intraductally with 100,000 control or 0.5% CSE-treated MCF 10A or MCF7 cells. Each mouse was injected at two sites. MCF10 A cells were injected into 3 mice and MCF7 cells into 2 mice for a total of 6 and 4 sites respectively. Two magnifications are shown. The xenografts were analyzed via immunoflourescence using a FITC antibody specific for human cytokeratin-18. **(C)** Growth of subcutaneous xenograft tumors from control and CSE-treated (0.5%) MCF7 cells. Each mouse (n=5) was injected at one site. Mean ± SD of five replicates are shown. **(D)** H&E staining of sections of lung and liver containing metastatic colonies from three representative NSG immunodeficient mice subcutaneously injected with 750,000 MCF7 cells treated with 0.5% CSE. Two additional mice were analyzed by gross pathology for a total n=5.

MCF 10A cells are not able to form tumors in immunodeficient mice, and are thus neither malignant nor tumorigenic. Based on our *in vitro* results, we hypothesized that treatment with CSE might drive these cells to become more invasive or pre-malignant. To investigate this scenario, we used an intraductal transplantation model originally developed to study ductal carcinoma in situ (DCIS). In this model, cancer cells are injected through the nipple, into the primary mammary duct, which allows in situ analysis of intraductal growth and/or invasion through the basement membrane into the stroma [18]. MCF 10A cells treated with 0.5% CSE for 46 weeks or mock treated were injected into the primary inguinal mammary ducts of 8-week-old female immunodeficient mice (NSG). The mammary fat pads were harvested after three months and labeled with an antibody for human cytokeratin-18 to identify the injected human cells. Untreated MCF 10A cells did not appear to colonize or grow in the ducts; however, colonies of CSE-treated MCF 10A cells were found within the ducts up to 3 months post-injection (Figure 3A). We then investigated if CSE could further increase the invasiveness of MCF7 breast cancer cells. Because these cells are tumorigenic, and grow much faster than MCF 10A, we harvested the mammary glands seven days after intraductal injection. At that time, untreated MCF7 cells had formed several intraductal masses that appeared to remain within the confines of the ducts. In contrast, MCF7 cells treated with CSE for 21 weeks had invaded through the duct and had formed colonies in the stroma (Figure 3B). Since treatment with CSE had clearly increased the invasiveness of MCF7 cells, we investigated if tumorigenesis and metastasis of MCF7 cells were also affected using a subcutaneous xenograft model. Transplanted MCF7 cells treated with 0.5% CSE for 18 weeks resulted in smaller tumors than mock-treated cells (Figure 3C); however, these smaller tumors were associated with metastases in the lungs of all animals and isolated` cells were found in the liver of at least one animal (Figure 3D). The three mice shown are representative, and two additional mice injected with CSE treated cells were analyzed by gross pathology for a total of 5. In contrast, we did not observe metastases from untreated MCF7 cells, suggesting that cigarette smoke may have favored the expansion of a highly metastatic subpopulation of MCF7 cells. Although MCF10A cells had exhibited increased intraductal survival and colonization after treatment with CSE, these cells did not produce subcutaneous tumors even after 43 weeks of exposure to CSE.

### CSE causes changes in stem cell markers in MCF 10A and MCF7 cells

**Figure 4:**
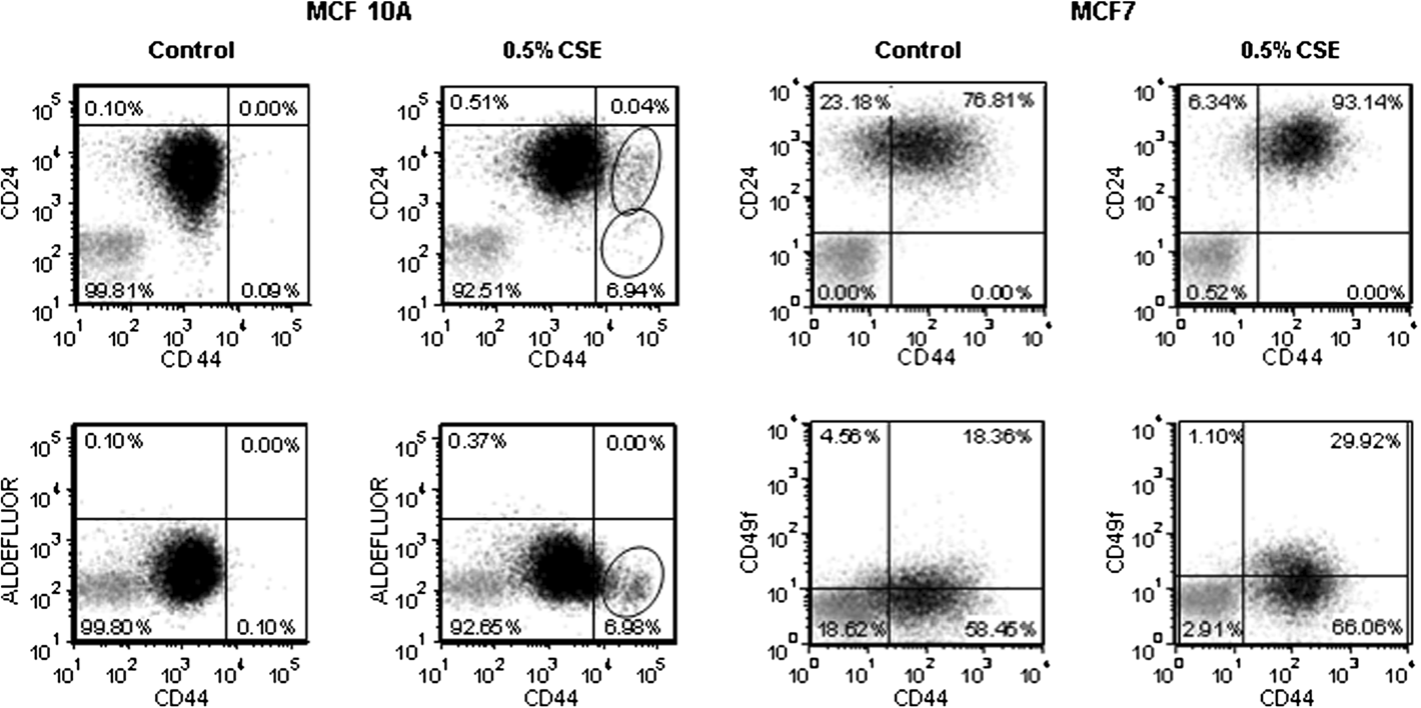
**CSE causes changes in stem cell markers in mammary epithelial cells and breast cancer cells.** FACS analysis of MCF 10A cells treated with CSE for 30 weeks and MCF7 cells treated with CSE for 17 weeks. Cells labeled with isotype control antibodies, or incubated with DEAB (negative control) are shown in grey. Cells labeled with specific antibodies, or by aldefluor reaction are in shown in black and the percentage of cells in each quadrant is shown. MCF 10A: quadrants were established to include 99.9% of CD24^+^/CD44^+^, or ALDEFLUOR^+^/CD44^+^ mock treated cells; cells with increased CD44 positivity (circled) are concentrating in the lower right quadrants after CSE treatment, indicating loss of CD24 positivity and ALDEFLUOR signal. MCF7: quadrants were established to include 99% of signal from isotype antibody (negative); mock treated MCF7 cells consist of a mixed population of CD44^+^ and CD44^-^ cells, uniformly CD24^+^; CSE treatment caused a shift to the CD24^+^/CD44^+^ quadrant, and an increase in CD49f^+^ cells.

Self renewal is a critical component of tumorigenesis [11]. Thus, we analyzed how CSE affects the distribution of specific cell surface markers that are associated with tumor initiation and self renewal, specifically ALDH1 activity, high CD44/low CD24, CD49f and CD133 [19-21]. FACS analysis showed a sharp change in the distribution of CD44 and CD24 in CSE-treated MCF 10A and MCF7 cells. Most MCF 10A cells are CD44^+^/CD24^+^, but after exposure to 0.5% CSE, at least two cell populations with substantially increased CD44 and lower ALDH activity emerged. In one of these populations the expression of CD24 was particularly low (Figure 4, left panels). In MCF10A cells, the distribution of CD49f was virtually unaffected by treatment with CSE, and the cells appeared uniformly CD49f^+^ (not shown). A small number of CD133^+^ cells that were entirely distinguishable from the CD44hi cells were present in MCF 10A cells treated with 0.5% CSE, while untreated cells were CD133^-^ (not shown). MCF7 cells are a mixed population of CD44^+^ and CD44^-^ cells, but are uniformly CD24^+^ (Figure 4, right panels). CSE treatment caused a shift to the CD24^+^/CD44^+^ quadrant, (76.8% to 93.1%) and an increase of CD49f positivity in a portion of the CD44^+^ cells (18.4% to 29.9%). These results indicate that chronic low-dose exposure to cigarette smoke can alter cellular distributions of markers associated with self-renewal and stem-like properties.

### Exposure of mammary epithelial cells to CSE affects the expression of genes associated with EMT and tumorigenesis

**Figure 5:**
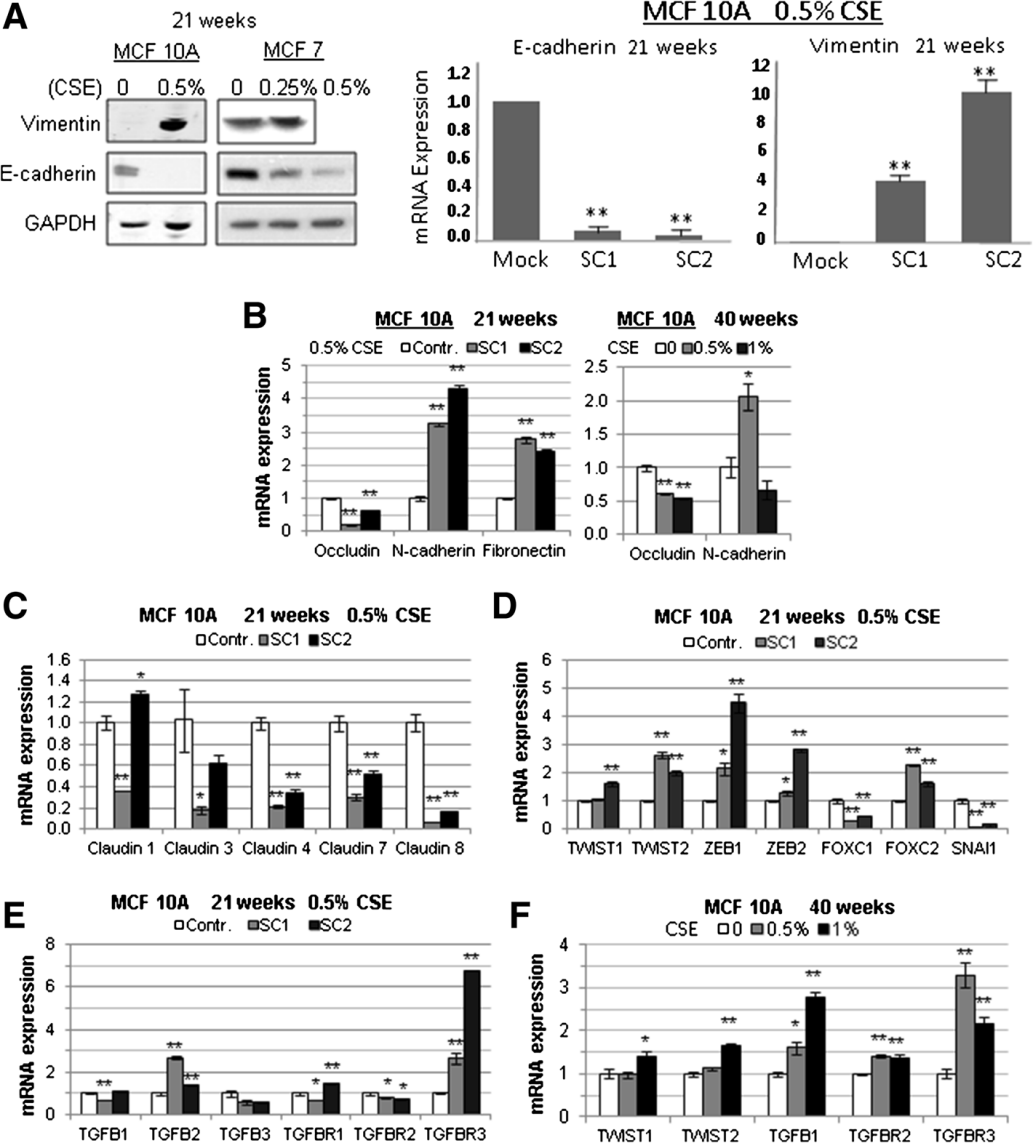
**Exposure of mammary epithelial cells to CSE leads to gene expression changes indicative of EMT. (A**, left panel**)** Western Blot showing upregulation of Vimentin and downregulation of E-cadherin in MCF10A and MCF7 cells exposed to CSE for 21 weeks. **(A**, right panel**)** qRT PCR analysis showing downregulation of e-cadherin mRNA and upregulation of Vimentin mRNA in two MCF10A subclones after 21 weeks of exposure to 0.5% CSE. **(B)** qRT PCR analysis showing downregulation of occludin and upregulation of N-cadherin and fibronectin in two MCF10A subclones at 21 and 40 weeks of exposure. **(C-F)** qRT PCR analysis of EMT genes in MCF 10A mammary epithelial cells exposed to 0.5% CSE for 21 weeks (clones SC1 and SC2) or 0.5-1.0% CSE for 40 weeks (without subcloning). Data in bar graphs are mean ± standard deviation of 3 replicates; *P<0.01, **P<0.001.

**Figure 6:**
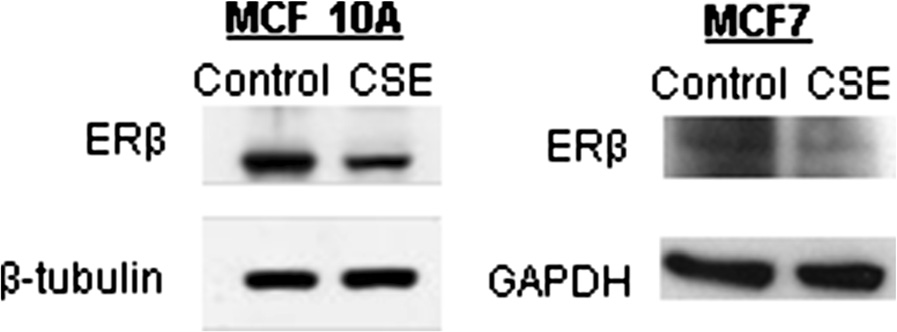
**Exposure of mammary epithelial cells or breast cancer cells to CSE causes downregulation of ERβ.** Western blot analysis of ERβ in MCF 10A mammary epithelial cells, and MCF7 breast cancer cells. Whole cell lysates were homogenized in high salt RIPA buffer and proteins were separated via 3-18% NuPage Tris-Acetate gels, transferred to PVDF and immunblotted with anti-ERβ. Enhanced chemiluminescence was used for visualization.

Two clones, designated SC1 and SC2 were isolated from MCF 10A cells exposed to 0.5% CSE for 13 weeks and expanded in the same concentration of CSE for 8 additional weeks, at which time total RNA was isolated for microarray and qPCR analysis. In addition, protein lysates and genomic DNA were prepared from independent samples treated with CSE for 21 weeks. The microarray analysis identified 186 unique genes that were upregulated and 308 unique genes that were downregulated at least two fold in both MCF 10A CSE clones (Additional file 1: Table S1). The expression of selected genes was verified by qRT-PCR or Western blot analysis (Figure 5), focusing on those genes associated with the phenotypes that we had observed, namely EMT, invasion and metastasis. E-cadherin and vimentin, which are associated with an epithelial state [22,23], were downregulated and upregulated respectively in MCF10As as determined by Western blot analysis (left panel) and PCR (right panel) at 21 weeks (Figure 5A). Similar changes were observed by Western blot analysis for MCF7 cells; however, vimentin data is only shown for a 0.25% CSE treatment (Figure 5A, left panel). Decreases in occludin, and increases in N-cadherin and fibronectin, which are also associated with a mesenchymal state [22], were observed in MCF10A cells treated with CSE for 21 weeks (Figure 5B left panel). In addition, we observed dysregulation of occludin and N-cadherin in an independent RNA sample of MCF 10A cells treated with CSE for 40 weeks (Figure 5B, right panel). We also observed a general downregulation of keratins (Additional file 1: Table S1), which is another hallmark of EMT [22]. Several members of the claudin family of tight-junction proteins were downregulated (Figure 5C), which fits with the observed increased motility induced by CSE treatment. The EMT-promoting transcription factors *TWIST1*, *TWIST2*, *ZEB1*, *ZEB2*, and *FOXC2* were upregulated, while *FOXC1* and *SNAI1* (Snail) were downregulated by CSE in MCF 10A cells (Figure 5D). These transcription factors can be induced through TGF-β signaling [24,25], and we observed that TGF-β receptor I and III (*TGFBR1*, *TGFBR3*), and TGF-β2 (*TGFB2*) were upregulated in MCF 10A cells treated with CSE (Figure 5E). Some of these gene expression changes were significant in only one of the two CSE-treated MCF 10A clones indicating variability in the response to smoke exposure. Upregulation of *TWIST1* and *TWIST2*, as well as of *TGFBR3* was also observed in MCF 10A treated with CSE for 40 weeks, together with *TGFB1*, but not *TGFB2* (Figure 5F). Since exposure to cigarette smoke has been previously linked to epigenetic silencing in human cancer [26,27], we investigated if promoter methylation could be responsible for gene downregulation in our model. We used a DNA methylation array to estimate the proportion of methylated loci (beta-value) in MCF 10A cells treated with CSE for 21 weeks (Additional file 2: Table S2), focusing on sites located within promoter CpG islands. The beta value of one *occludin* probe increased from 0.11 to 0.50 after treatment with CSE, indicating a substantial increase in methylation. Similarly, the beta-value of one *claudin 1* site increased from 0.06 to 0.55. None of the other downregulated genes that we had validated up to this point were affected according to this analysis. However, we observed increased methylation of estrogen receptor beta (ERβ; 0.17 to 0.72), which can act as a tumor suppressor in the mammary epithelium [28]. Western blot analysis showed that the expression of ERβ was reduced in MCF 10A and MCF7 cells treated with CSE (Figure 6). Although our data are in agreement with other published reports and suggest that this receptor may be epigenetically repressed by cigarette smoke, we cannot discount alternative transcriptional mechanisms and processes related to protein degradation or decreased stability.

## Discussion

After many years of debate, there is now ample proof that tobacco smoke increases the risk of breast cancer. Multiple studies, including some published after the last Surgeon General Report [5] and IARC monographs on the subject [29] show that active and passive exposure to tobacco smoke increases the risk of breast cancer in both premenopausal and postmenopausal women [1-5]. With the epidemiological evidence now conclusive, the task remains to investigate the molecular mechanism by which exposure to tobacco smoke, either voluntary or involuntary, leads to increased breast cancer risk [5].

The response of breast epithelial cells and breast cancer cell to cigarette smoke has been previously examined [15,16,30,31], but these studies focused on short-term treatment (up to one week) while we have analyzed the effect of continuous long-term exposure. We demonstrated that chronic exposure to tobacco smoke in the form of CSE or CSC can alter the phenotype of mammary epithelial cells, promoting the acquisition of mesenchymal traits such as increased anchorage-independent growth, motility, invasion, and the expression of markers associated with self-renewal and tumor initiation. Numerous groups have demonstrated the emergence of a CD44^+^/CD24^-/low^ stem-like signature from CD44^+/low^/CD24^+^ cells upon the induction of an EMT phenotype characterized by loss of E-cadherin and gain of vimentin [11,32]. The CD44^+^/CD24^-/low^ phenotype has been consistently associated with self-renewing mammary epithelial cells, which are also more tumorigenic and basal-like than CD44^+^/CD24^+^ cells [33]. Similarly, we showed that treatment of MCF 10A cells with CSE leads to the emergence of a CD44^hi^/CD24^low^ population, and our *in vivo* experiments demonstrated that CSE-treated MCF 10A cells have increased survival and colonization ability. Although MCF 10A cells did not become malignant, treatment of the MCF7 cancer cell line led to increased metastatic potential, consistent with published evidence that the differentiation state of the cell of origin is a strong determinant of the cellular phenotype of the final transformed state [34]. Other studies in animal models have previously shown that tobacco smoke can increase the risk of metastasis from breast cancer, but this has been attributed mainly to smoking-induced inhibition of host antitumor immune defenses, or to damage of the host tissue [35,36]. In contrast, our data from *ex vivo* exposure followed by orthotopic or subcutaneous transplantation into mice indicate that tobacco smoke can directly affect the ability of breast epithelial cells to invade or metastasize, independent of other cigarette smoke effects on the host and stromal environment.

The phenotypical alterations induced by cigarette smoke were accompanied by multiple gene expression changes. We concentrated our analysis on genes associated with EMT, loss of tumor suppression and the acquisition of malignancy traits. Our data indicates that ERβ is epigenetically repressed by tobacco smoke, which is consistent with a recent study showing that methylation of ERβ is a frequent event in breast cancer [37]. Contrary to the better known and structurally similar ERα, ERβ does not induce mitogenic response and can reduce basal, hormone-independent cell proliferation [28]. ERβ is widely expressed in normal mammary epithelium, but frequently lost in breast cancer, where its presence generally correlates with better prognosis [28,38]. Knock down of ERβ in MCF 10A or MCF7 cells was shown to cause a significant growth increase of both cell types in a ligand-independent manner [38], while expression of exogenous ERβ in the receptor negative breast cancer cell line MDA-MB-231 inhibited proliferation [39]. Cigarette smoke also caused downregulation of claudin 1, 3, 4, 7, and 8. The claudins are integral components of tight junctions, and their expression in cancer appears to be tissue specific, with some claudins downregulated in certain tumors and upregulated in others [40]. A small subgroup of breast cancer has been identified as expressing low levels of claudins, and is referred to as the “claudin low” group [41-43]. Claudin low tumors represent 12-13% of breast cancers, are generally basal like, and overexpress EMT markers [41-43]. Mouse claudin-low tumors generated in a p53-null animal model were found to be markedly enriched in tumor-initiating cells [44]. Consistently, our claudin-low CSE-treated breast cells are more tumorigenic than untreated cells, and exhibit gene expression changes indicative of EMT, such as downregulation of E-cadherin and occludin, and upregulation of N-cadherin, fibronectin and vimentin. Downregulation of occludin can reduce cancer sensitivity to apoptogenic factors by modulating apoptosis-associated genes. In addition, occludin decreases cellular invasiveness and motility, thus its downregulation can potentially favor cancer metastasis [45]. The downregulation of occludin and claudin 1 [46] may also be the result of epigenetic regulation, since we have observed increased methylation at the promoter of these genes and in the case of claudin 1, the gene can be re-expressed with demethylating agents such as 5-azacytidine and decitabine [46]. CSE treatment upregulated *TGFBR3* and *TGFB2* in MCF 10A cells, which is consistent with the reported observation that endothelial cells undergoing EMT express *TGFBR3*, and TGFBR3-specific antisera can inhibit mesenchyme formation and migration [24]. Moreover, ectopic overexpression of *TGFBR3* in non-transforming ventricular endothelial cells conferred transformation in response to TGFB2 [24]. Since we observed upregulation of *TGFBR3* and *TGFB2* in MCF 10A cells that are undergoing EMT-like changes, but are not completely transformed by cigarette smoke, our results suggest that overexpression of these two genes by cigarette smoke may be a component of EMT that is not associated with transformation. Alternatively, this could be a very early event in transformation and cancer development. We also observed that the EMT-promoting transcription factors *TWIST1*, *TWIST2*, *ZEB1*, *ZEB2* and *FOXC2* were upregulated, while *FOXC1* and *SNAI1* (Snail) were downregulated by CSE. Except for the decreased *SNAI1*, these data are consistent with recent reports that the MDA-MB-231 and MDA-MB-435 basal B cell lines express higher levels of fibronectin, N-cadherin, *SNAI1* and *ZEB2*, and lower E-cadherin and *FOXC1* than the luminal epithelial cell line, MCF7 [25]. The same study showed that overexpression of *TWIST1*, as well as the EMT-promoting factor TGF-β1, consistently upregulates *ZEB1* and *ZEB2* and *FOXC2* in human mammary epithelial cells. Interestingly, TGF-β1 is up-regulated by TWIST1, but is not required for TWIST1-induced up-regulation of *FOXC2*, which occurs in mammary epithelial cells overexpressing *TWIST1* even in the presence of a TGF-β signaling inhibitor [25]. Taken together our observations in the MCF 10A breast epithelial cell line exposed to CSE are consistent with a model of EMT where TWIST drives the transition and upregulates *FOXC2*, *ZEB1* and *ZEB2*, with potential involvement of TGFβ signaling.

## Conclusions

Our results indicate that chronic, long-term exposure to cigarette smoke leads to a more aggressive and transformed phenotype in human mammary epithelial cells, and that the differentiation state of the cell at the time of exposure may be a critical determinant in the phenotype of the final transformed state. Non-malignant, human mammary epithelial cells (MCF 10A) exposed to cigarette smoke in the form of CSE survived intraductally in a mouse mammary gland many months beyond their normal capacity, and breast cancer cells which normally do not metastasize in mice (MCF7), formed metastatic colonies in the lung. All CSE-treated cell lines showed EMT-like behavior including increased anchorage-independent growth, increased motility and invasiveness, and we observed an increase in markers of self-renewing cells, along with accompanying gene expression changes indicative of EMT and malignancy.

## Methods

### Cell culture model of exposure to cigarette smoke

Cigarette smoke extract (CSE) was prepared weekly by burning 2 complete 1R3F cigarettes (Kentucky Tobacco Research and Development Center, Lexington, KY, USA) and drawing the smoke by vacuum into 10 ml of sterile PBS. CSE concentration was evaluated by measuring the optical density at 502.4 nm, and diluted to O.D. = 0.10±0.01 [47]. This solution was considered 100% CSE. The concentration of nicotine was evaluated by mass spectrometry as previously described [48]. The 100% CSE contained 253±22 μg/ml of nicotine which is equivalent to 0.2 cigarettes/ml. Cigarette smoke condensate (CSC) was purchased from Murty pharmaceuticals (Lexington, KY, USA) and is prepared by smoking a 1R3F cigarette on a smoking machine and collecting the particulate matter from the side stream smoke onto a filter for extraction with DMSO. Cell lines were purchased from ATCC (Manassas, VA, USA) and cultured with either CSE, or CSC refreshing the media and additives twice a week (see Additional file 3 for media formulation).

### *In vitro* transformation assays

Anchorage-independent growth was assessed by seeding the cells on soft agar (0.4% top layer, 0.8% bottom layer); and counting the colonies after 14 days using an inverted microscope and 0.005% crystal violet for staining. Cell migration and invasion was assessed in Boyden chambers using 8μm-pore inserts, with or without matrigel coating (BD Bioscience, San Jose, CA, USA) according to the manufacturer’s instructions.

### Animal studies

NOD-SCID and NSG mice were purchased from The Jackson Laboratory (Bar Harbor, ME, USA), and cared for in strict accordance with an approved Johns Hopkins ACUC protocol. Intraductal transplantation was performed as described previously [18]. Briefly, 10^5^ cells were injected in the mammary ducts of immunodeficient female NSG mice as 2 μL of single-cell suspension in PBS with 0.1% trypan blue, using a Hamilton syringe with a blunt-ended 1/2-inch 30-gauge needle. At the indicated times, mice were euthanized, and the mammary fat pads harvested and fixed in 10% neutral buffer formalin. For xenografts, CSE treated cells (10^6^) were subcutaneously injected as a 50 μl single-cell suspension in a 1:1 solution of media and BD Matrigel Matrix (BD Bioscience). At the indicated times, the mice were euthanized, and fixed by perfusion with PBS followed by 10% neutral buffer formalin for necropsy. Female mice receiving MCF7 cells were implanted with beeswax pellets containing 20 μg of estrogen one day before injection [49]. Paraffin embedded sections were analyzed by standard H&E staining, and by immunohistochemistry using a monoclonal antibody for human cytokeratin-18 (C1399, Sigma-Aldrich, St. Louis, MO, USA), and the Mouse on Mouse (M.O.M.) Fluorescein Kit (Vector Labs, Burlingame, CA, USA).

### Flow cytometry

Fluorescence-activated cell sorting (FACS) was performed on a BD Bioscience SLRII instrument. Cells were labeled using the ALDEFLUOR® kit (Stem Cell Technologies, Vancouver, BC, Canada), or the antibody conjugates listed in the Additional file 3.

### Analysis of gene expression and methylation

Microarray based gene expression and methylation analysis were performed at the microarray core of the SKCCC using the Agilent Human 44K expression array (Agilent Technologies, Santa Clara, CA, USA) and the Infinium Methylation27 Array (Illumina, Inc., San Diego, CA, USA) as previously described [50]. The data is deposited in the GEO database under accession number GSE42668. Quantitative Real-Time PCR analysis (qRT-PCR) was performed using a 7500 Real-Time PCR System, the High Capacity cDNA Reverse Transcription Kit, TaqMan Gene Expression Master Mix, and the TaqMan Gene Expression Assays listed in the Additional file 3 (Applied Biosystems, Foster City, CA, US). Western blot analysis was performed as previously described [50] using the antibodies listed in the Additional file 3.

## Competing interests

The authors declare that they have no competing interests.

## Authors’ contributions

FDC and VLF participated in the design and execution of the overall study. VLF, HL, and BVP carried out *in vitro* experiments. BG, KH, JS, and RB carried out *in vivo* mouse experiments. WW analyzed the microarray data. CB carried out the pathologic analysis. SBB and CAZ conceived the study and interpreted the results. FDC and CAZ wrote the paper and all authors read and approved the final manuscript.

## Supplementary Material

Additional file 1: Table S1 Summary of the results of the gene expression microarray analysis. Probes showing a difference in gene expression of 2-folds or more in at least one clone are shown. The complete data is available in the GEO database under accession number GSE42668. Click here for file

Additional file 2: Table S2 Summary of the results of the gene methylation microarray analysis. Probes showing an increase in beta-value of 0.2 or more in CSE-treated MCF10A cells are shown. The complete data is available in the GEO database under accession number GSE42668. Click here for file

Additional file 3 Cell culture media, TaqMan Gene Expression Assays, and antibodies (western blot and flow cytometry) used in this study. Click here for file
