# Supplementary material for: Cigarette smoke induces epithelial to mesenchymal transition and increases the metastatic ability of breast cancer cells
Source: Mol Cancer. 2013 Aug 6;12:90. doi: 10.1186/1476-4598-12-90 (PMC3750372; doi:10.1186/1476-4598-12-90)
Supplement: Additional file 3 — Cell culture media, TaqMan Gene Expression Assays, and antibodies (western blot and flow cytometry) used in this study. [file 1476-4598-12-90-S3.doc]

**Supplemental Materials and Methods**

**Cell culture media**

MCF 10A and MCF-12A were grown in a 1:1 mixture of Dulbecco's modified Eagle's medium and Ham's F12 medium supplemented with 20 ng/ml Human epidermal growth factor, 100 ng/ml cholera toxin, 0.01 mg/ml bovine insulin, 500 ng/ml hydrocortisone, and 5% horse serum. MCF7 were grown in Eagle's Minimum Essential Medium supplemented with 0.01 mg/ml bovine insulin and 10% fetal bovine serum (MCF7).

**TaqMan Gene Expression Assays** (Applied Biosystems, Foster City, CA, USA)

| occludin | Hs00170162_m1 |  | SNAI1 | Hs00195591_m1 |
| --- | --- | --- | --- | --- |
| fibronectin 1 | Hs01549976_m1 |  | FOXC1 | Hs00270951_s1 |
| N-cadherin | Hs00983056_m1 |  | FOXC2 | Hs00559473_s1 |
| TGFβ1 | Hs00998130_m1 |  | ZEB1 | Hs00232783_m1 |
| TGFβ2 | Hs00234244_m1 |  | ZEB2 | Hs00207691_m1 |
| TGFβ3 | Hs01086000_m1 |  | claudin 1 | Hs00221623_m1 |
| TGFβ receptor 1 | Hs00610320_m1 |  | claudin 3 | Hs00265816_s1 |
| TGFβ receptor 2 | Hs00234253_m1 |  | claudin 4 | Hs00976831_s1 |
| TGFβ receptor 3 | Hs01114253_m1 |  | claudin 7 | Hs00600772_m1 |
| TWIST1 | Hs00361186_m1 |  | claudin 8 | Hs00273282_s1 |
| TWIST2 | Hs02379973_s1 |  |  |  |

**Antibodies for western blot**

| vimentin | V9 | Thermo scientific, Waltham, MA, USA |
| --- | --- | --- |
| E-cadherin | 36 | BD Bioscience, San Jose, CA, USA |
| ERβ | H-150 | Santa Cruz Biotechnology, Inc., Dallas, Texas, USA |
| GAPDH | 2275 | Trevigen, Gaithersburg, MD, USA |

**Antibodies for flow cytometry**

| CD24 | FITC | BD Bioscience, San Jose, CA, USA |
| --- | --- | --- |
| CD44 | APC | BD Bioscience, San Jose, CA, USA |
| CD44 | AF700 | BD Bioscience, San Jose, CA, USA |
| CD24 | PE/Cy7 | Biolegend, San Diego, CA, USA |
| CD49f | PerCP/Cy5.5 | Biolegend, San Diego, CA, USA |
| CD133 | APC | Miltenyi Biotec, Auburn, CA, USA |
| CD133 | PE | Miltenyi Biotec, Auburn, CA, USA |
